# Chloridobis{2-[(dimethyl­amino)­meth­yl]phen­yl}anti­mony(III)

**DOI:** 10.1107/S1600536809041890

**Published:** 2009-10-17

**Authors:** Marian Olaru, Sorin Roşca, Ciprian I. Raţ, Cristian Silvestru

**Affiliations:** a Universitatea Babeş-Bolyai, Facultatea de Chimie şi Inginerie Chimicã, 11 Arany Janos, 400028 Cluj-Napoca, Romania

## Abstract

In the title compound, [Sb(C_9_H_12_N)_2_Cl], the Sb atom adopts a Ψ-trigonal-bipyramidal geometry. The two 2-[(dimethyl­amino)­methyl]­phenyl ligands are coordinated asymmetrically to the Sb atom. The carbon atoms of one of the ligands are disordered over sets of sites with equal occupancy, resulting in two conformational isomers in the crystal. The Sb—C and Sb—N distances in the ordered ligand are: 2.153 (4) and 3.326 (5) Å, respectively. The corresponding distances in the disordered ligand are: 2.103 (5)/2.188 (5) and 2.454 (3) Å, respectively. The structure displays intra­molecular C—H⋯Cl hydrogen bonding.

## Related literature

For the structure of the perdeuterobenzene solvate of the title compound, see: Carmalt *et al.* (1997 ▶). For anti­mony(III) compounds with 2-[(dimethyl­amino)­methyl]­phenyl substituents, see: Kamepalli *et al.* (1996 ▶); Tokunaga *et al.* (2000*a* ▶,*b* ▶); Breunig *et al.* (2003 ▶); Opris *et al.* (2003 ▶, 2004 ▶, 2009 ▶); Sharma *et al.* (2004 ▶).
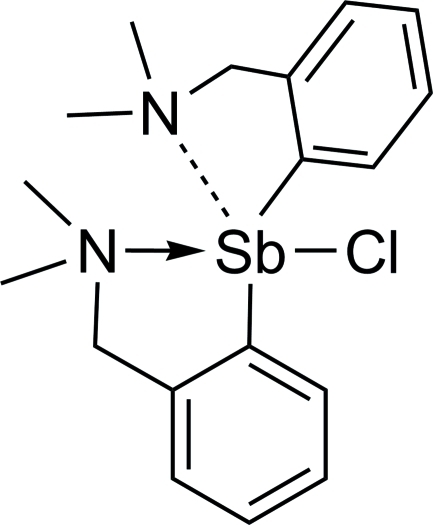


## Experimental

### 

#### Crystal data

[Sb(C_9_H_12_N)_2_Cl]
*M* *_r_* = 425.60
Triclinic, 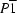

*a* = 9.289 (7) Å
*b* = 9.367 (7) Å
*c* = 12.888 (10) Å
α = 98.073 (13)°
β = 103.611 (13)°
γ = 116.819 (12)°
*V* = 932.6 (13) Å^3^
*Z* = 2
Mo *K*α radiation
μ = 1.62 mm^−1^
*T* = 297 K
0.33 × 0.31 × 0.27 mm

#### Data collection

Bruker SMART APEX CCD area-detector diffractometer
Absorption correction: multi-scan (*SADABS*; Bruker, 2000 ▶) *T*_min_ = 0.617, *T*_max_ = 0.669
10065 measured reflections
3801 independent reflections
3530 reflections with *I* > 2σ(*I*)
*R*_int_ = 0.027

#### Refinement

*R*[*F* ^2^ > 2σ(*F* ^2^)] = 0.035
*wR*(*F* ^2^) = 0.078
*S* = 1.13
3801 reflections
263 parameters
H-atom parameters constrained
Δρ_max_ = 0.78 e Å^−3^
Δρ_min_ = −0.45 e Å^−3^

### 

Data collection: *SMART* (Bruker, 2000 ▶); cell refinement: *SAINT-Plus* (Bruker, 2001 ▶); data reduction: *SAINT-Plus*; program(s) used to solve structure: *SHELXS97* (Sheldrick, 2008 ▶); program(s) used to refine structure: *SHELXL97* (Sheldrick, 2008 ▶) and *WinGX* (Farrugia, 1999 ▶); molecular graphics: *DIAMOND* (Brandenburg, 2009 ▶); software used to prepare material for publication: *publCIF* (Westrip, 2009 ▶).

## Supplementary Material

Crystal structure: contains datablocks I, global. DOI: 10.1107/S1600536809041890/pv2214sup1.cif

Structure factors: contains datablocks I. DOI: 10.1107/S1600536809041890/pv2214Isup2.hkl

Additional supplementary materials:  crystallographic information; 3D view; checkCIF report

## Figures and Tables

**Table 1:** Hydrogen-bond geometry (Å, °)

*D*—H⋯*A*	*D*—H	H⋯*A*	*D*⋯*A*	*D*—H⋯*A*
C6—H6⋯Cl1	0.93	2.65	3.291 (7)	127
C6*A*—H6*A*⋯Cl1	0.93	2.74	3.353 (7)	125

**Fig. 1.**
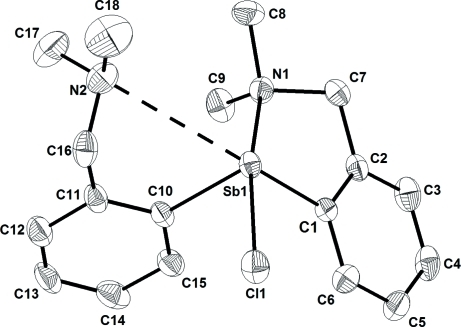
Graphical representation of the molecular structure of RN1,SN2-1. Hydrogen atoms were omitted for clarity. Displacement ellipsoids are drawn at 25% probability.

**Fig. 2.**
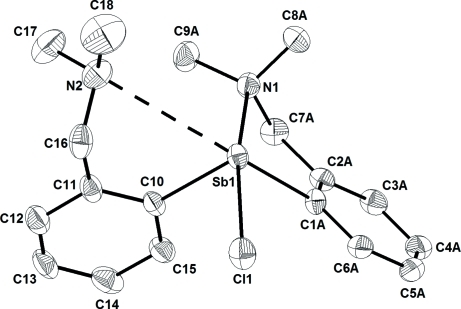
Graphical representation of the molecular structure of SN1,SN2-1. Hydrogen atoms were omitted for clarity. Displacement ellipsoids are drawn at 25% probability.

## supplementary crystallographic information

### Comment

The molecular structure of the perdeuterated benzene solvate of the title compound, (I).C_6_D_6_, was first determined by Carmalt *et al*. (1997). The existence of different enantiomers in the crystal structures of bis[2-(dimethylaminomethyl)phenyl]organoantimony(III) bromide and iodide has been reported (Opris *et al.*, 2003).

In the structure of (I), there are two dimethylaminomethylphenyl ligands that are asymmetrically coordinated to the Sb atom; the Sb atom adopts a Ψ-trigonal-bipyramidal geometry. The ligand containing nitrogen atom N1 was found to be disordered over two positions with s.o.f. of 0.501 (6) (Fig. 1), and 0.499 (6) (Fig. 2) thus resulting in two conformational isomers. Intramolecular hydrogen bonds are present between the chlorine atom and hydrogen atom in position 6 of the organic substituent containing the N1 atom (Table 1).

Bond distances and bond angles in (I) are similar to those reported for the perdeuteraded benzene solvate of the title compound (Carmalt *et al.*, 1997).

### Experimental

The title compound was prepared according to a previously described method (Carmalt *et al.*, 1997). Colourless crystals were obtained from a solution in chloroform by slow evaporation of the solvent.

### Refinement

The organic group containing N1 was found disordered over two positions. The components of the disorder were refined using a second free-variable. The phenyl groups were refined as rigid groups.

All nonhydrogen atoms were treated anisotropically. Hydrogen atoms were placed in calculated positions with isotropic thermal parameters set at 1.2 times the carbon atoms directly attached for aromatic and methylene hydrogen atoms, and 1.5 for hydrogen atoms of the methyl groups. The position of the hydrogen atoms of the methyl groups was calculated from the electron density.

### Crystal data

**Table:**

[Sb(C_9_H_12_N)_2_Cl]	*Z* = 2
*M**_r_* = 425.60	*F*(000) = 428
Triclinic, *P*1	*D*_x_ = 1.516 Mg m^−^^3^
Hall symbol: -P 1	Mo *K*α radiation, λ = 0.71073 Å
*a* = 9.289 (7) Å	Cell parameters from 3906 reflections
*b* = 9.367 (7) Å	θ = 2.5–24.4°
*c* = 12.888 (10) Å	µ = 1.62 mm^−^^1^
α = 98.073 (13)°	*T* = 297 K
β = 103.611 (13)°	Blocks, colourless
γ = 116.819 (12)°	0.33 × 0.31 × 0.27 mm
*V* = 932.6 (13) Å^3^	

### Data collection

**Table:**

Bruker SMART APEX CCD area-detector diffractometer	3801 independent reflections
Radiation source: fine-focus sealed tube	3530 reflections with *I* > 2σ(*I*)
graphite	*R*_int_ = 0.027
φ and ω scans	θ_max_ = 26.4°, θ_min_ = 2.5°
Absorption correction: multi-scan (*SADABS*; Bruker, 2000)	*h* = −11→11
*T*_min_ = 0.617, *T*_max_ = 0.669	*k* = −11→11
10065 measured reflections	*l* = −16→16

### Refinement

**Table:**

Refinement on *F*^2^	Primary atom site location: structure-invariant direct methods
Least-squares matrix: full	Secondary atom site location: difference Fourier map
*R*[*F*^2^ > 2σ(*F*^2^)] = 0.035	Hydrogen site location: inferred from neighbouring sites
*wR*(*F*^2^) = 0.078	H-atom parameters constrained
*S* = 1.13	*w* = 1/[σ^2^(*F*_o_^2^) + (0.035*P*)^2^ + 0.2481*P*] where *P* = (*F*_o_^2^ + 2*F*_c_^2^)/3
3801 reflections	(Δ/σ)_max_ = 0.001
263 parameters	Δρ_max_ = 0.78 e Å^−^^3^
0 restraints	Δρ_min_ = −0.45 e Å^−^^3^

### Special details

**Table:**

Geometry. All s.u.'s (except the s.u. in the dihedral angle between two l.s. planes) are estimated using the full covariance matrix. The cell s.u.'s are taken into account individually in the estimation of s.u.'s in distances, angles and torsion angles; correlations between s.u.'s in cell parameters are only used when they are defined by crystal symmetry. An approximate (isotropic) treatment of cell s.u.'s is used for estimating s.u.'s involving l.s. planes.
Refinement. Refinement of *F*^2^ against ALL reflections. The weighted *R*-factor *wR* and goodness of fit *S* are based on *F*^2^, conventional *R*-factors *R* are based on *F*, with *F* set to zero for negative *F*^2^. The threshold expression of *F*^2^ > 2σ(*F*^2^) is used only for calculating *R*-factors(gt) *etc*. and is not relevant to the choice of reflections for refinement. *R*-factors based on *F*^2^ are statistically about twice as large as those based on *F*, and *R*- factors based on ALL data will be even larger.

### Fractional atomic coordinates and isotropic or equivalent isotropic displacement parameters (Å^2^)

**Table:**

	*x*	*y*	*z*	*U*_iso_*/*U*_eq_	Occ. (<1)
C1	0.7468 (8)	0.4324 (9)	0.6493 (5)	0.047 (6)	0.501 (6)
C2	0.6306 (6)	0.2626 (10)	0.6213 (6)	0.048 (2)	0.501 (6)
C3	0.6832 (9)	0.1477 (7)	0.5996 (7)	0.065 (3)	0.501 (6)
H3	0.6055	0.034	0.5808	0.078*	0.501 (6)
C4	0.8521 (10)	0.2026 (8)	0.6058 (7)	0.065 (4)	0.501 (6)
H4	0.8873	0.1257	0.5913	0.078*	0.501 (6)
C5	0.9683 (8)	0.3724 (8)	0.6339 (7)	0.067 (2)	0.501 (6)
H5	1.0813	0.4091	0.6381	0.081*	0.501 (6)
C6	0.9156 (8)	0.4873 (6)	0.6556 (6)	0.058 (2)	0.501 (6)
H6	0.9934	0.601	0.6744	0.069*	0.501 (6)
C7	0.4477 (9)	0.2067 (9)	0.6097 (6)	0.055 (2)	0.501 (6)
H7A	0.3864	0.2057	0.5372	0.065*	0.501 (6)
H7B	0.3913	0.0952	0.6181	0.065*	0.501 (6)
C8	0.2676 (10)	0.3037 (11)	0.6608 (8)	0.074 (3)	0.501 (6)
H8A	0.2416	0.3183	0.5877	0.111*	0.501 (6)
H8B	0.2619	0.3844	0.712	0.111*	0.501 (6)
H8C	0.1859	0.1933	0.6589	0.111*	0.501 (6)
C9	0.4842 (13)	0.2998 (11)	0.8043 (7)	0.069 (3)	0.501 (6)
H9A	0.391	0.1953	0.8018	0.104*	0.501 (6)
H9B	0.495	0.3882	0.859	0.104*	0.501 (6)
H9C	0.5886	0.2963	0.8238	0.104*	0.501 (6)
C1A	0.7522 (8)	0.4295 (8)	0.6416 (5)	0.042 (5)	0.499 (6)
C2A	0.6821 (9)	0.2798 (10)	0.6685 (5)	0.048 (2)	0.499 (6)
C3A	0.7282 (10)	0.1620 (8)	0.6387 (7)	0.056 (3)	0.499 (6)
H3A	0.6813	0.0618	0.6568	0.067*	0.499 (6)
C4A	0.8444 (10)	0.1940 (7)	0.5819 (7)	0.068 (4)	0.499 (6)
H4A	0.8752	0.1152	0.562	0.081*	0.499 (6)
C5A	0.9145 (7)	0.3438 (8)	0.5549 (6)	0.060 (2)	0.499 (6)
H5A	0.9922	0.3652	0.5169	0.072*	0.499 (6)
C6A	0.8684 (7)	0.4615 (6)	0.5847 (6)	0.0510 (19)	0.499 (6)
H6A	0.9153	0.5617	0.5667	0.061*	0.499 (6)
C7A	0.5589 (10)	0.2447 (9)	0.7299 (7)	0.057 (2)	0.499 (6)
H7A1	0.6209	0.2862	0.8093	0.068*	0.499 (6)
H7A2	0.4835	0.1251	0.7115	0.068*	0.499 (6)
C8A	0.3298 (10)	0.2411 (10)	0.5935 (7)	0.064 (2)	0.499 (6)
H8A1	0.2438	0.1354	0.5954	0.096*	0.499 (6)
H8A2	0.3839	0.2235	0.5421	0.096*	0.499 (6)
H8A3	0.2773	0.3044	0.5702	0.096*	0.499 (6)
C9A	0.3800 (12)	0.3423 (11)	0.7886 (7)	0.070 (3)	0.499 (6)
H9A1	0.3175	0.3997	0.7735	0.105*	0.499 (6)
H9A2	0.4715	0.4034	0.8587	0.105*	0.499 (6)
H9A3	0.3043	0.2327	0.7915	0.105*	0.499 (6)
C10	0.7798 (4)	0.6853 (4)	0.8586 (3)	0.0469 (8)	
C11	0.7516 (5)	0.7975 (4)	0.9215 (3)	0.0555 (9)	
C12	0.8347 (6)	0.8557 (5)	1.0355 (3)	0.0677 (11)	
H12	0.818	0.9321	1.078	0.081*	
C13	0.9393 (6)	0.8043 (6)	1.0865 (3)	0.0768 (14)	
H13	0.9908	0.8421	1.1635	0.092*	
C14	0.9696 (5)	0.6964 (6)	1.0246 (4)	0.0748 (13)	
H14	1.0441	0.6632	1.0595	0.09*	
C15	0.8894 (5)	0.6365 (5)	0.9100 (3)	0.0606 (10)	
H15	0.9101	0.5631	0.8681	0.073*	
C16	0.6406 (6)	0.8609 (5)	0.8671 (4)	0.0683 (11)	
H16A	0.6536	0.9523	0.9217	0.082*	
H16B	0.6789	0.9041	0.8086	0.082*	
C17	0.3876 (7)	0.6916 (8)	0.9063 (5)	0.1067 (18)	
H17A	0.4441	0.6456	0.9508	0.16*	
H17B	0.2676	0.6111	0.8728	0.16*	
H17C	0.4023	0.7903	0.9526	0.16*	
C18	0.3679 (9)	0.7882 (9)	0.7445 (6)	0.133 (3)	
H18A	0.3749	0.8867	0.785	0.199*	
H18B	0.25	0.7014	0.7116	0.199*	
H18C	0.417	0.8127	0.687	0.199*	
N1	0.4519 (4)	0.3284 (4)	0.6993 (2)	0.0517 (7)	
N2	0.4613 (5)	0.7338 (5)	0.8199 (3)	0.0714 (9)	
Sb1	0.65485 (3)	0.59640 (3)	0.680735 (17)	0.04391 (10)	
Cl1	0.92046 (15)	0.84267 (12)	0.66663 (9)	0.0677 (3)	

### Atomic displacement parameters (Å^2^)

**Table:**

	*U*^11^	*U*^22^	*U*^33^	*U*^12^	*U*^13^	*U*^23^
C1	0.044 (12)	0.053 (13)	0.038 (9)	0.024 (11)	0.009 (9)	0.004 (8)
C2	0.056 (5)	0.042 (4)	0.040 (5)	0.026 (4)	0.009 (4)	0.003 (4)
C3	0.093 (8)	0.052 (6)	0.058 (7)	0.043 (6)	0.023 (6)	0.015 (4)
C4	0.097 (12)	0.063 (9)	0.057 (6)	0.061 (9)	0.020 (6)	0.013 (5)
C5	0.070 (6)	0.077 (6)	0.068 (6)	0.045 (5)	0.031 (5)	0.016 (5)
C6	0.062 (5)	0.061 (5)	0.059 (5)	0.035 (4)	0.026 (5)	0.022 (4)
C7	0.054 (5)	0.042 (4)	0.052 (4)	0.018 (3)	0.011 (3)	0.005 (3)
C8	0.045 (4)	0.063 (5)	0.090 (7)	0.015 (4)	0.018 (4)	0.008 (5)
C9	0.085 (7)	0.057 (5)	0.057 (5)	0.026 (5)	0.028 (5)	0.021 (4)
C1A	0.056 (14)	0.038 (11)	0.038 (9)	0.028 (10)	0.016 (9)	0.013 (7)
C2A	0.045 (5)	0.045 (5)	0.048 (6)	0.021 (4)	0.008 (4)	0.012 (4)
C3A	0.054 (5)	0.036 (4)	0.068 (8)	0.021 (4)	0.011 (5)	0.011 (4)
C4A	0.050 (8)	0.069 (10)	0.072 (7)	0.034 (7)	0.006 (5)	−0.003 (5)
C5A	0.047 (4)	0.057 (5)	0.070 (6)	0.024 (4)	0.022 (4)	0.005 (4)
C6A	0.045 (4)	0.053 (4)	0.053 (5)	0.024 (4)	0.013 (4)	0.014 (4)
C7A	0.060 (5)	0.049 (4)	0.064 (5)	0.025 (4)	0.024 (4)	0.025 (4)
C8A	0.057 (5)	0.048 (4)	0.066 (5)	0.016 (4)	0.014 (4)	0.004 (4)
C9A	0.076 (6)	0.070 (6)	0.063 (5)	0.028 (5)	0.040 (5)	0.020 (4)
C10	0.0482 (18)	0.0412 (18)	0.0354 (17)	0.0122 (15)	0.0117 (14)	0.0073 (14)
C11	0.061 (2)	0.0398 (18)	0.0454 (19)	0.0094 (17)	0.0221 (17)	0.0055 (15)
C12	0.066 (3)	0.055 (2)	0.052 (2)	0.008 (2)	0.025 (2)	0.0036 (19)
C13	0.067 (3)	0.081 (3)	0.0329 (19)	0.005 (2)	0.0127 (19)	−0.001 (2)
C14	0.060 (3)	0.086 (3)	0.056 (3)	0.025 (2)	0.004 (2)	0.022 (2)
C15	0.056 (2)	0.066 (2)	0.046 (2)	0.025 (2)	0.0093 (17)	0.0096 (18)
C16	0.101 (3)	0.048 (2)	0.066 (3)	0.041 (2)	0.040 (2)	0.0163 (19)
C17	0.100 (4)	0.128 (5)	0.122 (5)	0.066 (4)	0.067 (4)	0.039 (4)
C18	0.146 (6)	0.149 (6)	0.161 (7)	0.114 (5)	0.044 (5)	0.077 (5)
N1	0.0538 (17)	0.0515 (17)	0.0482 (17)	0.0244 (15)	0.0207 (14)	0.0115 (14)
N2	0.078 (2)	0.076 (2)	0.083 (3)	0.050 (2)	0.036 (2)	0.034 (2)
Sb1	0.05461 (16)	0.04224 (14)	0.03608 (14)	0.02685 (12)	0.01251 (10)	0.01049 (9)
Cl1	0.0829 (7)	0.0465 (5)	0.0674 (6)	0.0227 (5)	0.0340 (6)	0.0182 (5)

### Geometric parameters (Å, °)

**Table:**

C1—C2	1.39	C7A—H7A1	0.97
C1—C6	1.39	C7A—H7A2	0.97
C1—Sb1	2.103 (5)	C8A—N1	1.391 (8)
C2—C3	1.39	C8A—H8A1	0.96
C2—C7	1.496 (9)	C8A—H8A2	0.96
C3—C4	1.39	C8A—H8A3	0.96
C3—H3	0.93	C9A—N1	1.479 (9)
C4—C5	1.39	C9A—H9A1	0.96
C4—H4	0.93	C9A—H9A2	0.96
C5—C6	1.39	C9A—H9A3	0.96
C5—H5	0.93	C10—C15	1.369 (5)
C6—H6	0.93	C10—C11	1.393 (5)
C7—N1	1.484 (8)	C10—Sb1	2.153 (4)
C7—H7A	0.97	C11—C12	1.383 (5)
C7—H7B	0.97	C11—C16	1.496 (6)
C8—N1	1.563 (9)	C12—C13	1.348 (7)
C8—H8A	0.96	C12—H12	0.93
C8—H8B	0.96	C13—C14	1.368 (7)
C8—H8C	0.96	C13—H13	0.93
C9—N1	1.406 (8)	C14—C15	1.387 (6)
C9—H9A	0.96	C14—H14	0.93
C9—H9B	0.96	C15—H15	0.93
C9—H9C	0.96	C16—N2	1.446 (6)
C1A—C2A	1.39	C16—H16A	0.97
C1A—C6A	1.39	C16—H16B	0.97
C1A—Sb1	2.188 (5)	C17—N2	1.451 (6)
C2A—C3A	1.39	C17—H17A	0.96
C2A—C7A	1.484 (9)	C17—H17B	0.96
C3A—C4A	1.39	C17—H17C	0.96
C3A—H3A	0.93	C18—N2	1.448 (6)
C4A—C5A	1.39	C18—H18A	0.96
C4A—H4A	0.93	C18—H18B	0.96
C5A—C6A	1.39	C18—H18C	0.96
C5A—H5A	0.93	N1—Sb1	2.454 (3)
C6A—H6A	0.93	N2—Sb1	3.326 (5)
C7A—N1	1.537 (8)	Sb1—Cl1	2.5759 (18)
			
C2—C1—C6	120	C12—C11—C10	118.7 (4)
C2—C1—Sb1	117.2 (4)	C12—C11—C16	120.2 (4)
C6—C1—Sb1	122.8 (4)	C10—C11—C16	121.0 (3)
C3—C2—C1	120	C13—C12—C11	121.5 (4)
C3—C2—C7	121.1 (6)	C13—C12—H12	119.2
C1—C2—C7	118.9 (6)	C11—C12—H12	119.2
C4—C3—C2	120	C12—C13—C14	119.8 (4)
C4—C3—H3	120	C12—C13—H13	120.1
C2—C3—H3	120	C14—C13—H13	120.1
C5—C4—C3	120	C13—C14—C15	120.2 (4)
C5—C4—H4	120	C13—C14—H14	119.9
C3—C4—H4	120	C15—C14—H14	119.9
C6—C5—C4	120	C10—C15—C14	120.0 (4)
C6—C5—H5	120	C10—C15—H15	120
C4—C5—H5	120	C14—C15—H15	120
C5—C6—C1	120	N2—C16—C11	112.9 (3)
C5—C6—H6	120	N2—C16—H16A	109
C1—C6—H6	120	C11—C16—H16A	109
N1—C7—C2	106.2 (5)	N2—C16—H16B	109
N1—C7—H7A	110.5	C11—C16—H16B	109
C2—C7—H7A	110.5	H16A—C16—H16B	107.8
N1—C7—H7B	110.5	N2—C17—H17A	109.5
C2—C7—H7B	110.5	N2—C17—H17B	109.5
H7A—C7—H7B	108.7	H17A—C17—H17B	109.5
N1—C8—H8A	109.5	N2—C17—H17C	109.5
N1—C8—H8B	109.5	H17A—C17—H17C	109.5
N1—C8—H8C	109.5	H17B—C17—H17C	109.5
N1—C9—H9A	109.5	N2—C18—H18A	109.5
N1—C9—H9B	109.5	N2—C18—H18B	109.5
N1—C9—H9C	109.5	H18A—C18—H18B	109.5
C2A—C1A—C6A	120	N2—C18—H18C	109.5
C2A—C1A—Sb1	116.7 (4)	H18A—C18—H18C	109.5
C6A—C1A—Sb1	123.1 (4)	H18B—C18—H18C	109.5
C3A—C2A—C1A	120	C8A—N1—C9	137.0 (6)
C3A—C2A—C7A	119.5 (6)	C8A—N1—C9A	114.3 (6)
C1A—C2A—C7A	120.5 (6)	C9—N1—C9A	48.6 (5)
C2A—C3A—C4A	120	C8A—N1—C7	51.7 (5)
C2A—C3A—H3A	120	C9—N1—C7	114.1 (6)
C4A—C3A—H3A	120	C9A—N1—C7	143.2 (5)
C5A—C4A—C3A	120	C8A—N1—C7A	110.2 (5)
C5A—C4A—H4A	120	C9—N1—C7A	59.8 (5)
C3A—C4A—H4A	120	C9A—N1—C7A	107.8 (6)
C6A—C5A—C4A	120	C7—N1—C7A	61.0 (4)
C6A—C5A—H5A	120	C8A—N1—C8	54.1 (5)
C4A—C5A—H5A	120	C9—N1—C8	108.4 (6)
C5A—C6A—C1A	120	C9A—N1—C8	64.2 (6)
C5A—C6A—H6A	120	C7—N1—C8	104.2 (5)
C1A—C6A—H6A	120	C7A—N1—C8	145.7 (5)
C2A—C7A—N1	110.5 (5)	C8A—N1—Sb1	105.1 (4)
C2A—C7A—H7A1	109.5	C9—N1—Sb1	117.9 (4)
N1—C7A—H7A1	109.5	C9A—N1—Sb1	114.6 (4)
C2A—C7A—H7A2	109.5	C7—N1—Sb1	102.2 (3)
N1—C7A—H7A2	109.5	C7A—N1—Sb1	104.3 (3)
H7A1—C7A—H7A2	108.1	C8—N1—Sb1	109.2 (4)
N1—C8A—H8A1	109.5	C16—N2—C18	110.2 (4)
N1—C8A—H8A2	109.5	C16—N2—C17	111.2 (4)
H8A1—C8A—H8A2	109.5	C18—N2—C17	110.9 (5)
N1—C8A—H8A3	109.5	C1—Sb1—C10	96.9 (2)
H8A1—C8A—H8A3	109.5	C1—Sb1—C1A	2.6 (3)
H8A2—C8A—H8A3	109.5	C10—Sb1—C1A	98.77 (18)
N1—C9A—H9A1	109.5	C1—Sb1—N1	73.2 (2)
N1—C9A—H9A2	109.5	C10—Sb1—N1	89.75 (12)
H9A1—C9A—H9A2	109.5	C1A—Sb1—N1	75.0 (2)
N1—C9A—H9A3	109.5	C1—Sb1—Cl1	91.9 (2)
H9A1—C9A—H9A3	109.5	C10—Sb1—Cl1	87.61 (10)
H9A2—C9A—H9A3	109.5	C1A—Sb1—Cl1	90.3 (2)
C15—C10—C11	119.7 (3)	N1—Sb1—Cl1	164.43 (8)
C15—C10—Sb1	121.0 (3)	N2—Sb1—Cl1	110.65 (8)
C11—C10—Sb1	119.3 (3)		
			
C6—C1—C2—C3	0	C11—C16—N2—C18	−165.1 (4)
Sb1—C1—C2—C3	180.0 (5)	C11—C16—N2—C17	71.6 (5)
C6—C1—C2—C7	−177.0 (7)	C2—C1—Sb1—C10	106.9 (4)
Sb1—C1—C2—C7	3.0 (7)	C6—C1—Sb1—C10	−73.1 (4)
C1—C2—C3—C4	0	C2—C1—Sb1—C1A	−114 (7)
C7—C2—C3—C4	177.0 (7)	C6—C1—Sb1—C1A	66 (7)
C2—C3—C4—C5	0	C2—C1—Sb1—N1	19.3 (4)
C3—C4—C5—C6	0	C6—C1—Sb1—N1	−160.7 (4)
C4—C5—C6—C1	0	C2—C1—Sb1—Cl1	−165.3 (4)
C2—C1—C6—C5	0	C6—C1—Sb1—Cl1	14.7 (4)
Sb1—C1—C6—C5	180.0 (5)	C15—C10—Sb1—C1	4.3 (4)
C3—C2—C7—N1	144.3 (5)	C11—C10—Sb1—C1	−177.3 (3)
C1—C2—C7—N1	−38.6 (8)	C15—C10—Sb1—C1A	2.6 (4)
C6A—C1A—C2A—C3A	0	C11—C10—Sb1—C1A	−179.0 (3)
Sb1—C1A—C2A—C3A	175.2 (4)	C15—C10—Sb1—N1	77.3 (3)
C6A—C1A—C2A—C7A	179.6 (7)	C11—C10—Sb1—N1	−104.3 (3)
Sb1—C1A—C2A—C7A	−5.2 (7)	C15—C10—Sb1—Cl1	−87.3 (3)
C1A—C2A—C3A—C4A	0	C11—C10—Sb1—Cl1	91.1 (3)
C7A—C2A—C3A—C4A	−179.6 (7)	C2A—C1A—Sb1—C1	33 (7)
C2A—C3A—C4A—C5A	0	C6A—C1A—Sb1—C1	−152 (7)
C3A—C4A—C5A—C6A	0	C2A—C1A—Sb1—C10	74.1 (3)
C4A—C5A—C6A—C1A	0	C6A—C1A—Sb1—C10	−110.8 (4)
C2A—C1A—C6A—C5A	0	C2A—C1A—Sb1—N1	−13.3 (3)
Sb1—C1A—C6A—C5A	−174.9 (5)	C6A—C1A—Sb1—N1	161.8 (4)
C3A—C2A—C7A—N1	−148.5 (4)	C2A—C1A—Sb1—Cl1	161.7 (3)
C1A—C2A—C7A—N1	31.9 (8)	C6A—C1A—Sb1—Cl1	−23.2 (4)
C15—C10—C11—C12	−0.6 (5)	C8A—N1—Sb1—C1	−90.6 (5)
Sb1—C10—C11—C12	−179.0 (3)	C9—N1—Sb1—C1	88.5 (6)
C15—C10—C11—C16	176.5 (4)	C9A—N1—Sb1—C1	143.0 (5)
Sb1—C10—C11—C16	−1.9 (5)	C7—N1—Sb1—C1	−37.4 (4)
C10—C11—C12—C13	−1.1 (6)	C7A—N1—Sb1—C1	25.4 (4)
C16—C11—C12—C13	−178.2 (4)	C8—N1—Sb1—C1	−147.3 (5)
C11—C12—C13—C14	2.2 (6)	C8A—N1—Sb1—C10	172.1 (4)
C12—C13—C14—C15	−1.7 (7)	C9—N1—Sb1—C10	−8.7 (5)
C11—C10—C15—C14	1.1 (6)	C9A—N1—Sb1—C10	45.8 (5)
Sb1—C10—C15—C14	179.5 (3)	C7—N1—Sb1—C10	−134.6 (4)
C13—C14—C15—C10	0.1 (6)	C7A—N1—Sb1—C10	−71.8 (4)
C12—C11—C16—N2	−113.5 (4)	C8—N1—Sb1—C10	115.5 (4)
C10—C11—C16—N2	69.5 (5)	C8A—N1—Sb1—C1A	−88.7 (4)
C2—C7—N1—C8A	147.4 (8)	C9—N1—Sb1—C1A	90.4 (6)
C2—C7—N1—C9	−80.7 (7)	C9A—N1—Sb1—C1A	145.0 (5)
C2—C7—N1—C9A	−133.0 (9)	C7—N1—Sb1—C1A	−35.5 (4)
C2—C7—N1—C7A	−52.1 (6)	C7A—N1—Sb1—C1A	27.3 (4)
C2—C7—N1—C8	161.3 (6)	C8—N1—Sb1—C1A	−145.4 (4)
C2—C7—N1—Sb1	47.7 (6)	C8A—N1—Sb1—Cl1	−107.7 (5)
C2A—C7A—N1—C8A	74.7 (7)	C9—N1—Sb1—Cl1	71.4 (6)
C2A—C7A—N1—C9	−151.9 (8)	C9A—N1—Sb1—Cl1	126.0 (5)
C2A—C7A—N1—C9A	−159.9 (6)	C7—N1—Sb1—Cl1	−54.5 (5)
C2A—C7A—N1—C7	58.5 (6)	C7A—N1—Sb1—Cl1	8.3 (5)
C2A—C7A—N1—C8	130.0 (9)	C8—N1—Sb1—Cl1	−164.4 (4)
C2A—C7A—N1—Sb1	−37.7 (6)		

### Hydrogen-bond geometry (Å, °)

**Table:**

*D*—H···*A*	*D*—H	H···*A*	*D*···*A*	*D*—H···*A*
C6—H6···Cl1	0.93	2.65	3.291 (7)	127
C6A—H6A···Cl1	0.93	2.74	3.353 (7)	125
